# Medical Colleges

**Published:** 1919-06

**Authors:** 


### TEXAS MEDICAL JOURNAL

DR. F. E. DANIEL,
Founder.

Established July,
1885

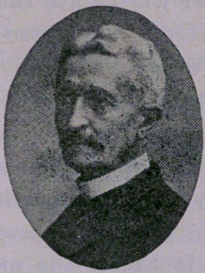

MRS. F. E. DANIEL,
Publisher and Managing
Editor.

VOL. XXXIV No. 12.
AUSTIN,
June, 1919

PUBLISHED MONTHLY.
Subscription:
$1.50

The publisher is not responsible for views of contributors.

ASSISTANT EDITORS:

DR. JOHN S. TURNER, Dallas, Texas.
DR. E. B. PARSONS, Palestine, Texas.
DR. A. P. HOWARD, Houston, Texas.
DR. JOE BECTON, Greenville, Texas.
DR. M. F. BLEDSOE, Port Arthur.
DR. J. H. FLORENCE, Tulsa, Oklahoma.
DR. J. H. REUSS, Cuero, Texas.

### EDITORIAL.

### Medical Colleges.

It was the intention of the Texas Medical Journal to devote a considerable amount of space in this issue to the interests of the medical colleges of the country. A carefully compiled list of the best medical schools was selected and an earnest effort was made to interest these with the view to awakening a general interest on the part of pre-medical students in the selection of medical colleges. Despite numerous inquiries and requests sent out there was such a lack of responses as to indicate that the heads of most medical schools are really very little concerned about increasing their attendance or enlarging the interest in medical education.

It is generally conceded that there will be a greater demand than ever, following the war, for well trained physicians and surgeons, and that with concerted effort on the part of the medical schools of the country this demand could easily be met. It is also agreed that the physicians of the future must be better equipped through professional training for the practice of the profession than the majority of the physicians of the past half century have been.

Are the medical colleges really prepared to give the needed training or not? Are their teaching forces keeping ahead of the profession or are they, in the fancied security of their positions, contented to run along the methods that were in use before the war? In other words, are the medical colleges progressive or have they gotten into a rut so deeply worn that they find it impossible to get out?

There is a vague suspicion that, in some cases at least, the instructional staffs of the medical schools are away behind the practitioner who comes into daily contact with the new problems that are constantly confronting the profession.

It would be interesting indeed to have a discussion of these questions from the standpoint of the medical schools as well as from the viewpoint of the man who is out in the field at work every day. The Texas Medical Journal is unable to decide whether or not the suspicion that exists with reference to medical schools is justified by the facts. What have the heads of the schools to say upon this subject? The columns of this publication are freely open to them for a presentation of their opinions upon this subject as well as to the views of the men in the practice.

Unless the medical schools can show that they are really at the head of the profession and that they are determined to keep abead, there is certainly going to arise a demand for a return to the old method of training under the direction of physicians who are in every-day practice. The building up of numerous laboratories and clinics in both private and public hospitals and sanitariums throughout the country will likely result in a desire on the part of students to get their training from these practical institutions rather than from such colleges as believe that their dignified standing is sufficient commendation to attract students.

In looking over the pages of the leading medical journals of the country it is noticeable that but few medical schools are bidding for patronage of the future physicians of the country through such publications. In this age students of every profession naturally turn toward those institutions that manifest most interest in them both before and after they get into the schools.

